# Fellow-Workers and Their Doings

**Published:** 1914-05-02

**Authors:** 


### ROUND THE WORLD.

### Fellow-Workers and their Doings.

[The Editor Invites Early Information and Contributions Marked "Fellow-Workers."]

The interest that has lately developed in connection with the treatment of pulmonary tuberculosis by inducing pneumothorax is both remarkable and noteworthy; there is even, as Dr. Claude Lillingston pointed out in The Hospital recently, a special international association for studying the method, with a journal publishing its reports. Among those who have carried out original work in this connection must be mentioned Dr. F. C. Coley, of Newcastle-on-Tyne, who has recently devised an improved type of apparatus for carrying out the operation for inducing an artificial pneumothorax. Dr. Coley, although a Durham M.D., is also a Guy's man and M.R.C.S., L.R.C.P., who has for many years practised at Newcastle, where he holds several important hospital and sanatorium appointments.

---

Mr. Harold A. Kisch, M.B., F.R.C.S., has attempted to make it possible for the nose, throat, and ear surgeon to determine with a greater degree of scientific accuracy than has hitherto been attained how far there be obstruction in the nasal passages without a patient being objectively aware that such is the case. To this end he has invented a rhinomanometer, which, as he states in his account of the instrument to the *British Medical Journal* (April 4), enables the expert to measure the extent to which the air passages are narrowed without loss of time or elaborate examination. Mr. Kisch, who is a member of the staffs of the Central London Throat and Ear and the Royal Ear Hospitals, considers that slight obstruction, the presence of which is not easy to ascertain, has an important bearing on the causation of chronic deafness in many instances.

---

Dr. A. D. Edwards, the medical officer of health for Bournemouth, will in all probability achieve a most beneficial result by his endeavours to get growing-pains recognised as a serious sign of impaired health in children. As the late Dr. Cheadle, of St. Mary's, used to insist, indefinite joint and limb pains in early life commonly indicate a rheumatic tendency which may already have led to the beginnings of a cardiac lesion, an opinion that was backed up by his authority as a pioneer in the investigation of the rheumatic disorders of childhood. Nevertheless, this point is not as generally appreciated as it well might be, considering its suspected bearing on later life, and Dr. Edwards, who is a former student of both University College, Cardiff, and the London Hospital, rightly attaches importance to the explanation to parents of the true meaning of "growing-pains."

---

Mr. Donald Duff, who has been appointed lecturer on surgery in the Glasgow Western Medical School, thus continues to achieve distinction in the city where he first elected to study medicine. Mr. Duff holds numerous important hospital posts in Glasgow, and, besides possessing the ordinary triple qualification, is an F.R.C.S. of Edinburgh.

---

Dr. Andrew Wilson, M.D. Glasg., who has been appointed lecturer on ophthalmology in the same school, is surgeon to the Glasgow Eye Infirmary, and succeeds Dr. James Hinshelwood, M.D., F.F.P.S. Glasg., who has for many years been known in Scotland as a prominent ophthalmologist.

Drs. F. H. Thiele and Dennis Embleton, who have lately published a method of increasing the accuracy and delicacy of the Wassermann reaction (*Lancet*, April 11), have, of course, carried out this important development at University College Hospital, where they are respectively lecturer and assistant lecturer in bacteriology. It is claimed that this method has the obvious advantages of rapidity and simplicity, as well as being easily controlled, whilst a further point in its favour is that no vivisection licence is required to carry out any stage of the process.

---

Dr. George Alexander Williamson, M.A., M.D., C.M., D.P.H., the new lecturer on tropical medicine at Aberdeen, returns to his Alma Mater on taking up this appointment. After qualifying in 1893, Dr. Williamson held various resident posts in Scotland, and ultimately gave his attention to the branch of medical work in connection with which his researches have brought him distinction. Going to Cyprus, he gained considerable experience of malaria and other "fevers" as district medical officer of Larnaca, and has written a good deal on the diseases endemic to that island. Dr. Williamson took the D.T.M. of Liverpool in 1906, and has lately been residing in Aberdeen, where he has taken great interest in the medical volunteer movement.

---

Mr. T. W. Letchworth, M.B., F.R.C.S., will be able to give the Hospital of St. John and St. Elizabeth, where he has lately joined the staff as ophthalmic surgeon, the benefit of much clinical experience gained at two leading eye hospitals—the Royal Westminster Ophthalmic and the Royal Eye—and also at the Marylebone General Dispensary. Mr. Letchworth is an old Cambridge and "Bart.'s" man.

---

Dr. Morden Carthew, to whom permission has lately been granted to wear the insignia of the third class of the Order of the Crown of Siam, is the first assistant medical officer at Bangkok; he is an M.D., Ch.B. of Edinburgh and a D.P.H. of Dublin.

---

Dr. Courtenay Yorke, the new honorary laryngologist to the Liverpool Hospital for Consumption and Diseases of the Chest, is an M.D. of Liverpool and an F.R.C.S. Eng. Dr. Yorke won several University distinctions, and after qualification held the post of house surgeon at the Northern Hospital, Liverpool.

---

Dr. Charlton Bastian, M.D., F.R.C.P., who, in a communication to the *Times*, has recently suggested that micrococci and bacilli are possibly evolved out of inorganic substances under conditions still prevalent on this planet, is a distinguished member of the consulting staff at both University College Hospital and the Queen Square Hospital for Paralysis and Epilepsy. For some years this veteran scientist has fought for his theories in face of the most obstinate opposition, and, however sceptical the observer of his experiment may be, Dr. Bastian's quiet courtesy and patience, as he explains his views of the possibility of spontaneous generation, rarely fail to impress.

